# Metal based donepezil analogues designed to inhibit human acetylcholinesterase for Alzheimer’s disease

**DOI:** 10.1371/journal.pone.0211935

**Published:** 2019-02-20

**Authors:** Md. Junaid, Nazrul Islam, Md. Kamal Hossain, M. Obayed Ullah, Mohammad A. Halim

**Affiliations:** 1 Department of Pharmaceutical Sciences, North South University, Dhaka, Bangladesh; 2 Department of Chemistry, Jahangirnagar University, Savar, Dhaka, Bangladesh; 3 Division of Computer-Aided Drug Design, The Red-Green Research Centre, Dhaka, Bangladesh; Weizmann Institute of Science, ISRAEL

## Abstract

Among neurodegenerative disorders, Alzheimer’s disease (AD) is one of the most common disorders showing slow progressive cognitive decline. Targeting acetylcholinesterase (AChE) is one of the major strategies for AD therapeutics, as cholinergic pathways in the cerebral cortex and basal forebrain are compromised. Herein, we report the design of some copper and other metal based donepezil derivatives, employing density functional theory (DFT). All designed compounds are optimized at the B3LYP/SDD level of theory. Dipole moments, electronic energie, enthalpies, Gibbs free energies, and HOMO-LUMO gaps of these modified compounds are also investigated in the subsequent analysis. The molecules were then subjected to molecular docking analysis with AChE to study the molecular interactions broadly. Ensemble based docking and molecular dynamics (MD) simulations of the best candidates were also performed. Docking and MD simulation reveal that modified drugs are more potent than unmodified donepezil, where Trp86, Tyr337, Phe330 residues play some important roles in drug-receptor interactions. According to ensemble based docking, D9 shows greater binding affinity compared to the parent in most conformations obtained from protein data bank and MD simulation. In addition, it is observed that the π- π stacking with the residues of Trp86, Tyr337, Tyr341, Tyr124 and Trp286 may be required for strong ligand binding. Moreover, ADME/T analysis suggests that modified derivatives are less toxic and have improved pharmacokinetic properties than those of the parent drug. These results further confirm the ability of metal-directed drugs to bind simultaneously to the active sites of AChE and support them as potential candidates for the future treatment of Alzheimer’s disease.

## Introduction

In recent decades, Acetylcholinesterase (AChE) has become a major interest in Alzheimer’s disease (AD) research. AD, a neural degenerative disorder, is characterized by accumulation of extracellular and vascular amyloid in the brain [1–3]. In brief, inhibitors of AChE enhance the acetylcholine (ACh) level and also sustain the duration of neurotransmitter action. AChE is also proposed to play an important role in Aβ-aggregation during plaque formation [4]. Research from the last decades explain the pathology of AD; in which, one strategy suggested that a decrease in AChE production at synaptic junction highly correlated with the onset of AD progression [5–7]. Estimations regarding AD showed that there are 35.6 million people with dementia worldwide as of 2010, and every 20 years this number is projected to double reaching 65.7 million in 2030 and 115.4 million in 2050 [8]. Several AChE inhibitors such as galantamine, donepezil, tacrine and rivastigmine are available for AD therapy, known to inhibit AChE, however, they are effective to treat mild to moderate AD only [9]. These drugs showed a non-selective profile along with limited efficacy, adverse cholinergic side effects in the periphery, poor bioavailability, and hepatotoxicity, though around 40–70% patients benefit from AChE inhibitors [10].

The crystal structure of AChE resolved by X-ray crystallographic technique contains two main binding sites, including the catalytic active site (CAS), which is formed by serine, histidine and glutamate, and the peripheral anionic site (PAS) connected by a deep, hydrophobic gorge [11]. Among the drugs targeting AChE, donepezil and other bifunctional inhibitors also may span the AChE gorge [12]. The detailed interactions analysis suggested that this drug has an exclusive orientation, extending from the CAS (bottom near Trp86) to the PAS (top near Trp286), along the active-site gorge. These studies established a structural baseline for improved inhibitor design of next-generation derivatives [13–15].

The use of metals in drug design has recently gained interest by the success of the anticancer drug, cisplatin [16,17]. Recently, drugs based on metal complexes are used as therapeutic agents (*e*.*g*., Pt, Au, and Ru) in the treatment of malignant diseases, including several types of cancers [18,19]. Therefore, in this study, a main focus was to design metal based analogues of donepezil by adding Cu^2+^ as a metal to improve its activity and efficacy. Theoretical work is conducted and validated using density functional theory (DFT), molecular docking, and molecular dynamics (MD) simulation studies. Moreover, some other metals (such as Fe, Co, Zn and Ni) are also incorporated with donepezil similar to the best copper based derivative.

## Methods

### Designing and optimization of ligands

The molecules were drawn on the BIOVIA Drawer. 3D structures were then generated by fully optimizing with DFT, employing Becke’s exchange functional combining Lee, Yang, and Parr’s (LYP) correlation functional [20,21]. As all designed compounds were modified with metal atoms, the SDD (Stuttgart/Dresden) basis set was used [22]. After optimization, subsequent vibrational frequency calculations were performed to confirm that the stationary points corresponded to minima on the potential energy surface. Electronic energies, enthalpies, Gibbs free energies, dipole moments, and partial charge analysis of each compound were also investigated. Hardness and softness of all compounds were determined from the energies (ε) of frontier HOMOs and LUMOs. Considering the Parr and Pearson interpretation [23] of DFT and Koopmans theorem [24], hardness (η), and softness (S) of the drugs were calculated according to the following equation.

$$\eta =\frac{\left(\varepsilon_{HOMO}-\varepsilon_{LUMO}\right)}{2}$$

$$s=\frac{1}{\eta }$$

### Molecular docking analysis

The three-dimensional crystal structure of recombinant human AChE (PDB ID: **4ey7**) was retrieved in pdb format from the protein data bank [25]. The model was then subjected to energy minimization using the steepest descent and conjugate gradient technique to eliminate bad contacts of protein atoms. Computations were carried out *in vacuo* with the GROMOS 96 43B1 parameters set, with implementation using the Swiss-PDB Viewer. For docking analysis, AutodockVina was employed and AutoDock Tools (ADT) of the MGL software package was used to convert pdb into a pdbqt format to input protein and ligands. The size of grid box in AutoDockVina was kept at 58.81735, 61.2066, and 72.8273 respectively for X, Y, Z. AutodockVina was implemented through the shell script provided by AutoDockVina developers. The binding affinity of ligand was observed by kcal/mole as a unit for a negative score [26].

### Molecular dynamics simulation

To validate the predictions from docking studies, MD simulation was performed using the NAMD [27] software, version 2.9. In this study, the CHARMM force field [28] was utilized, as it is widely applied to describe the macromolecular system. The Transferable Intermolecular Potential3 Points (TIP3P) water model was used by adding Cl^-^ and/or Na^+^ ions, where the total solvent molecules, 20109, have a density of 1.012 gm/cm^3^. A periodic boundary condition was employed to perform the simulation, where the box size used was 82.4×85.0×98.8Å^3^. Following the steepest descent energy minimization, equilibration of 100 steps was performed by NPT ensemble. Using Langevin Dynamics for constant temperature, full-system periodic electrostatics were maintained using the Particle Mesh Ewald (PME)[29]. Consistently Nose-Hoover Langevin piston [30,31] was used for constant pressure dynamics and SHAKE was used to keep all bonds involving hydrogen atoms at their equilibrium values. Finally, the full system was subjected to MD production run at 300 K for 25 ns in the NVT ensemble. The MD trajectories were saved every 50 ps for analysis.

### Ensemble based molecular docking

To further clarify the results of docking predictions, we used an ensemble based docking method, where two different approaches were employed to obtain different conformations from AChE. In the first approach, different crystallographic conformations of AChE were retrieved from protein data bank, PDB IDs: 1b41, 1f8u, 1vzj, 2x8b, 3lii, 4bdt, 4ey6, 4ey8, 4moe, 4pqe, 5foq, 5fpq, 5hf5, 5hf6, 5hf8, 5hf9, 5hfa. In the second approach, conformers were taken from the 25 ns MD simulation (PDB ID: **4ey7**) at every 1 ns of the 25 ns MD simulation. Against these conformers, the compounds donepezil, D8, D9 and D10 were subjected for docking using the same protocol discussed above in the methods section.

### Pharmacokinetic parameters study

To check the pharmacokinetic parameters and toxicity of the modified compounds and parent compound, the admetSAR server was utilized. We have utilized the admetSAR online database to evaluate the pharmacokinetics parameters related to drug absorption, metabolism and toxicity of the parent drug and its designed analogues [32]. Using structure similarity search methods, admetSAR predicts the latest and most comprehensive manually curated data for diverse chemicals associated with known ADME/T profiles.

For ADMET analysis, the admetSAR program was used in which 96,000 unique compounds with 45 kinds of ADMET-associated properties, proteins, species, or organisms have been carefully curated from a large number of diverse literatures. Although it is quite difficult to verify all of these compounds and to know whether this program included metal-based drugs or not, we used well known Pt-based cisplatin and carboplatin as well as metal-based drugs approved in the FDA and in clinical trials as test candidates to verify our metal-based donepezil drugs.

## Results and discussions

### Strategies and optimization of designed analogue

The new analogues of donepezil used in this study were designed according to the structural properties of the active site of AChE. As described above, among the two binding sites of AChE, the peripheral anionic site plays a significant role in ligand reorganization and allosteric activators [33,34]. The stabilization of the substrates binding on this site is largely π-cation interaction, while choline ester substrate specificity is mediated partly by Phe295 and Phe297 [35]. From detailed analysis of enzyme-inhibitor complexes, it appeared that the indole ring of Trp286 was involved in direct interaction with several inhibitors, showing a number of interaction modes including stacking, aromatic-aromatic, and π-cation, according to the nature of the ligands [36–38]. Furthermore, the active site of AChE forms electrostatic interactions with the substrates, as all of the amino acids were distributed with a large dipole moment. Information from the above studies, therefore, motivated us to design new analogues of donepezil, by increasing their electronegativity and the non-covalent interaction capacity between the aromatic rings.

**Fig 1:**
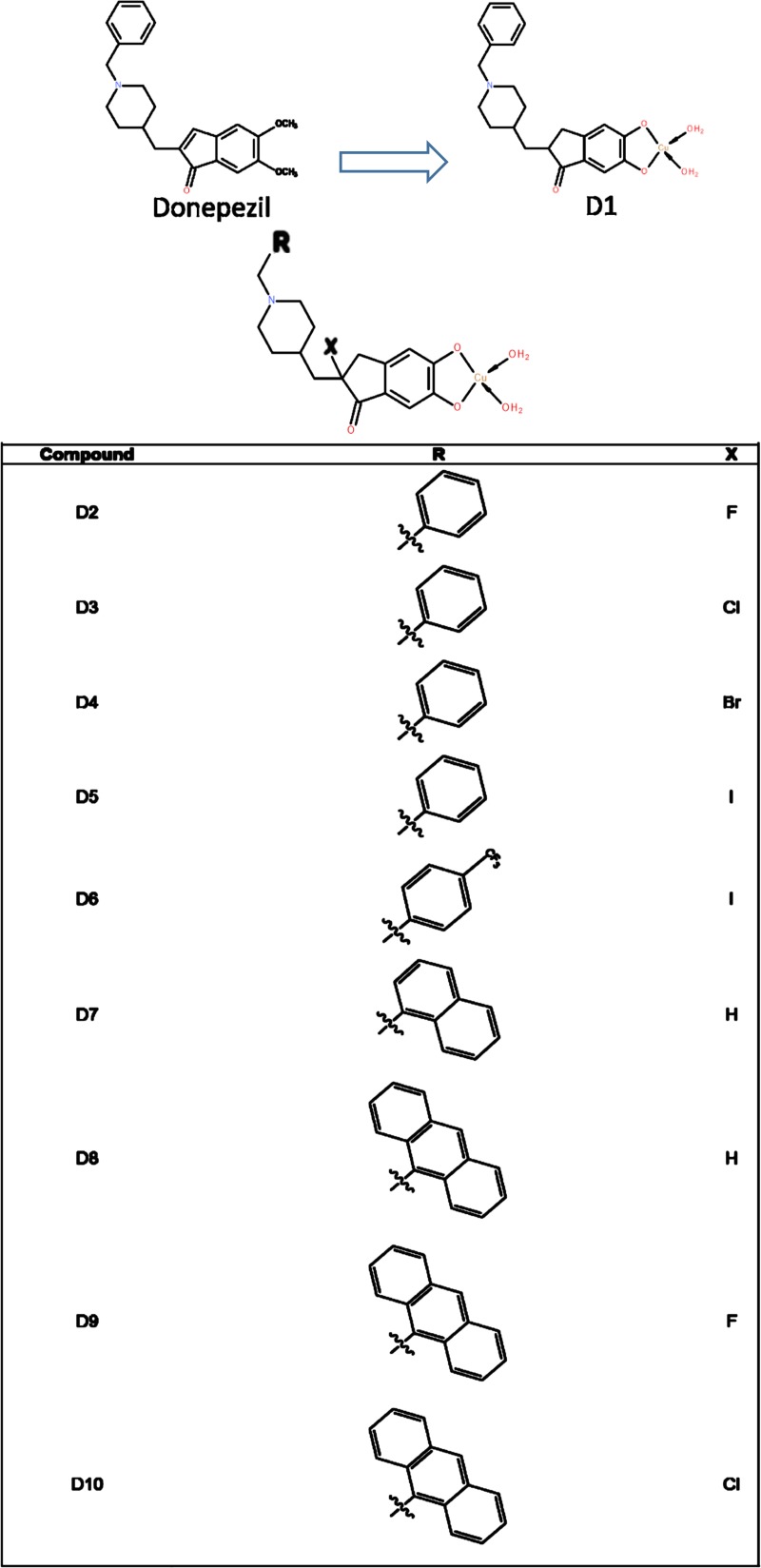
The design of new analogues based on the potent, first generation molecule, donepezil. Here, D1 was designed from donepezil, while others (D2 to D10) are based on the basic structure of D1.

As shown in **Fig 1**, ten analogues (D1-D10) were designed by modifying donepezil (D), which may react with [CuCl_2_(H_2_O)_2_] affording the probable mononuclear copper complexes [Cu(D)n(H_2_O)_2_]. There were also several additional modifications in D2-D10. D2-D5 were modified by the addition of F (D2), Cl (D3), Br (D4), and I (D5) atoms in the 2,3-dihydroindene ring portion, respectively. In contrast, D6 was designed by corresponding with D5 while modifications occurred only in the attached benzene ring, *i*.*e*., benzene ring with CF_3_ group. In D7 and D8, attached benzene ring of the parent structure was replaced by naphthalene and anthracene rings, respectively, with no halogen modification; however, replacement of H with F and Cl atoms at the 2,3-dihydroindene ring portion of D8 results new analogues D9 and D10, respectively.

**Fig 2:**
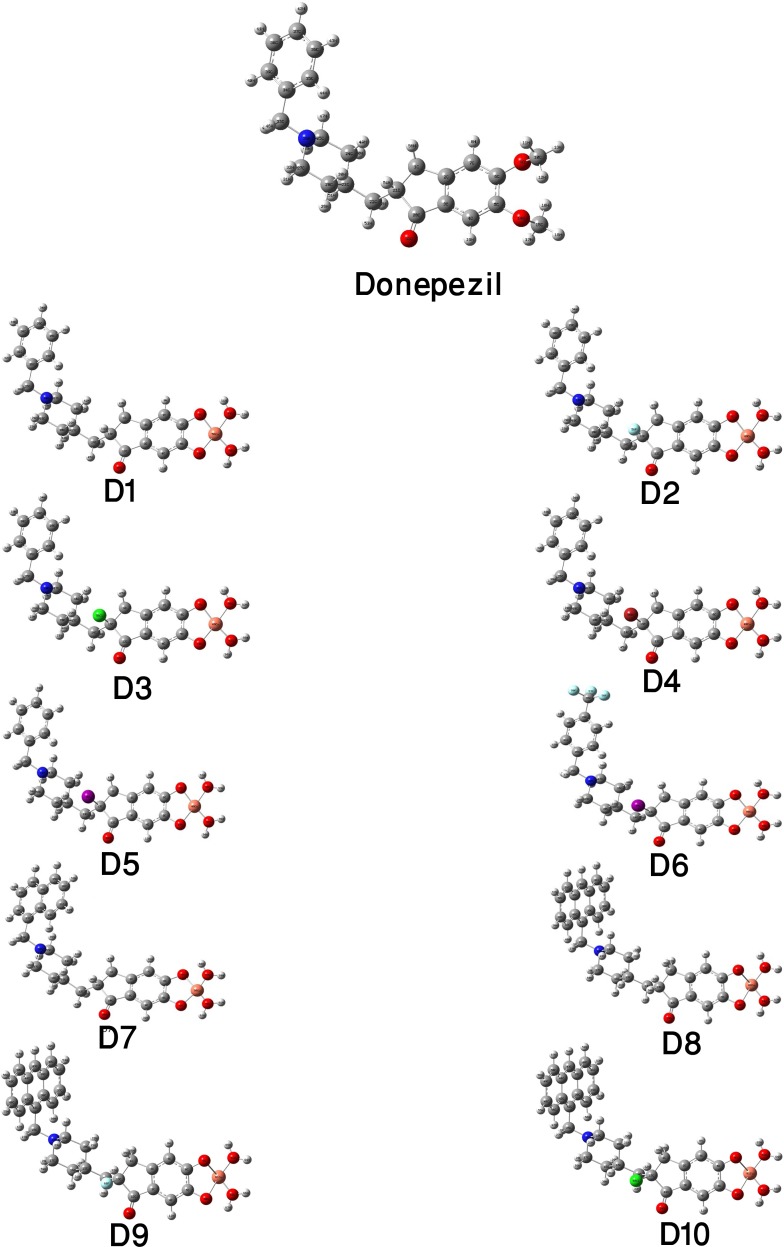
Most stable optimized structures of all designed analogues along with their parent molecule, donepezil. All analogues were optimized in the gas phase at B3LYP/SDD level in Gaussian 09.

As the conformational features of a molecule critically influences its physical and chemical properties, all of designed compounds along with parent compound, donepezil, were subjected to full geometry optimization using DFT. Table 1 illustrates the stoichiometry, electronic energy, enthalpy, Gibbs free energy and dipole moment of the compounds and the optimized structures are depicted in **Fig 2**.

**Table 1:** The stoichiometry, electronic energy, enthalpy, Gibbs free energy (in Hartree), and dipole moment (Debye) of donepezil and its designed analogues.

Name	Stoichiometry	Electronic Energy	Enthalpy	Gibbs Free Energy	Dipole Moment (Debye)
Donepezil	C_24_H_29_NO_3_	-1204.88	-1204.88	-1204.97	2.575
D1	C_22_H_27_CuNO_5_(2)	-1482.22	-1482.22	-1482.31	11.349
D2	C_22_H_26_CuFNO_5_(2)	-1581.48	-1581.48	-1581.57	13.547
D3	C_22_H_26_ClCuNO_5_(2)	-1941.80	-1941.80	-1941.90	13.328
D4	C_22_H_26_BrCuNO_5_(2)	-1495.00	-1495.00	-1495.10	13.299
D5	C_22_H_26_CuINO_5_(2)	-1493.04	-1493.03	-1493.13	13.070
D6	C_23_H_25_CuF_3_INO_5_(2)	-1830.08	-1830.08	-1830.19	12.787
D7	C_26_H_29_CuNO_5_(2)	-1635.79	-1635.79	-1635.89	11.360
D8	C_30_H_31_CuNO_5_(2)	-1789.35	-1789.35	-1789.45	11.821
D9	C_30_H_30_CuFNO_5_(2)	-1888.61	-1888.61	-1888.71	13.596
D10	C_30_H_30_ClCuNO_5_(2)	-2248.93	-2248.93	-2249.04	13.346

According to the **Table 1**, it is clear that modifications on donepezil significantly influenced the structural properties of the compounds in terms of energy, partial charge distribution, and dipole moment. The highest energy, enthalpy, and Gibbs free energy was observed for D10, while D9 showed the highest dipole moment of 13.596 Debye, representing high polarity in nature. It is important to note that incorporation of the–CF_3_ group in D5 significantly reduced the dipole moment, as can be seen in D6; however, D1, D7, D8 showed low dipole moments of 13.596 Debye due to the lack of halogens.

### Analysis of frontier molecular orbitals

**Fig 3:**
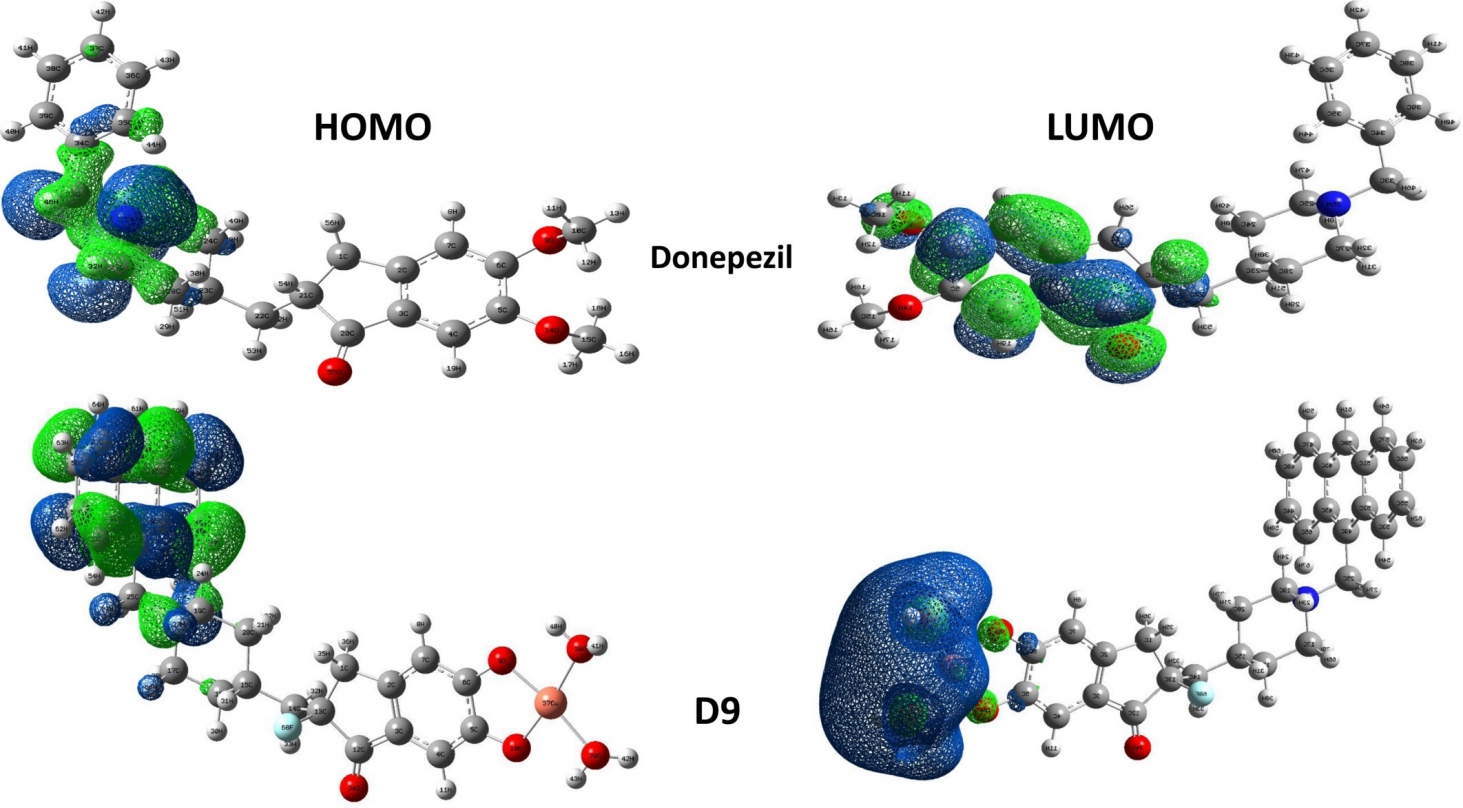
Molecular orbital distribution plots of HOMO and LUMO in the ground state of D9 analogue and donepezil at DFT/SDD level of theory in the gas phase.

The frontier molecular orbitals are the most important orbitals in a molecule and they are considered to characterize the chemical reactivity and kinetic stability. These frontier molecular orbitals are known as the highest occupied molecular orbital (HOMO) and the lowest unoccupied molecular orbital (LUMO). **Table 2** represents the values of orbital energies, along with the two global chemical descriptors, hardness and softness, which are also calculated for all compounds. The highest softness was observed for D8. D8 also showed the lowest HOMO-LUMO gap and hardness, indicating that the molecule is more reactive than other compounds, according to Pearson *et al*. [39,40]. In **Fig 3**, the HOMO plot of compound D9 showed that the electrons were localized on the upper part of the piperidine ring, while the LUMO plot showed that the electrons were localized at modified Cu regions only.

**Table 2:** Energy of HOMOs, LUMO, gap, hardness and softness (all units are in Hartree) of the donepezil and its designed analogues.

Molecules	*ε*_*HOMO-1*_	*ε*_*HOMO*_	*ε*_*LUMO*_	*Gap*	*η* (Hardness)	*S* (Softness)
Donepezil	-0.23073	-0.21374	-0.04412	0.16962	0.08481	11.7911
D1	-0.19606	-0.18662	-0.05973	0.12689	0.063445	15.7617
D2	-0.19405	-0.19230	-0.06407	0.12823	0.064115	15.5970
D3	-0.19398	-0.19335	-0.06368	0.12967	0.064835	15.4238
D4	-0.19498	-0.19336	-0.06365	0.12971	0.064855	15.4190
D5	-0.19536	-0.19256	-0.06432	0.12824	0.06412	15.5958
D6	-0.20900	-0.19490	-0.06797	0.12693	0.063465	15.7567
D7	-0.19577	-0.18635	-0.05953	0.12682	0.06341	15.7703
D8	-0.19064	-0.18695	-0.06296	0.12399	0.061995	16.1303
D9	-0.19382	-019036	-0.06383	0.12653	0.063265	15.8065
D10	-0.19344	-0.19093	-0.06358	0.12735	0.063675	15.70475

### Molecular docking analysis

**Fig 4:**
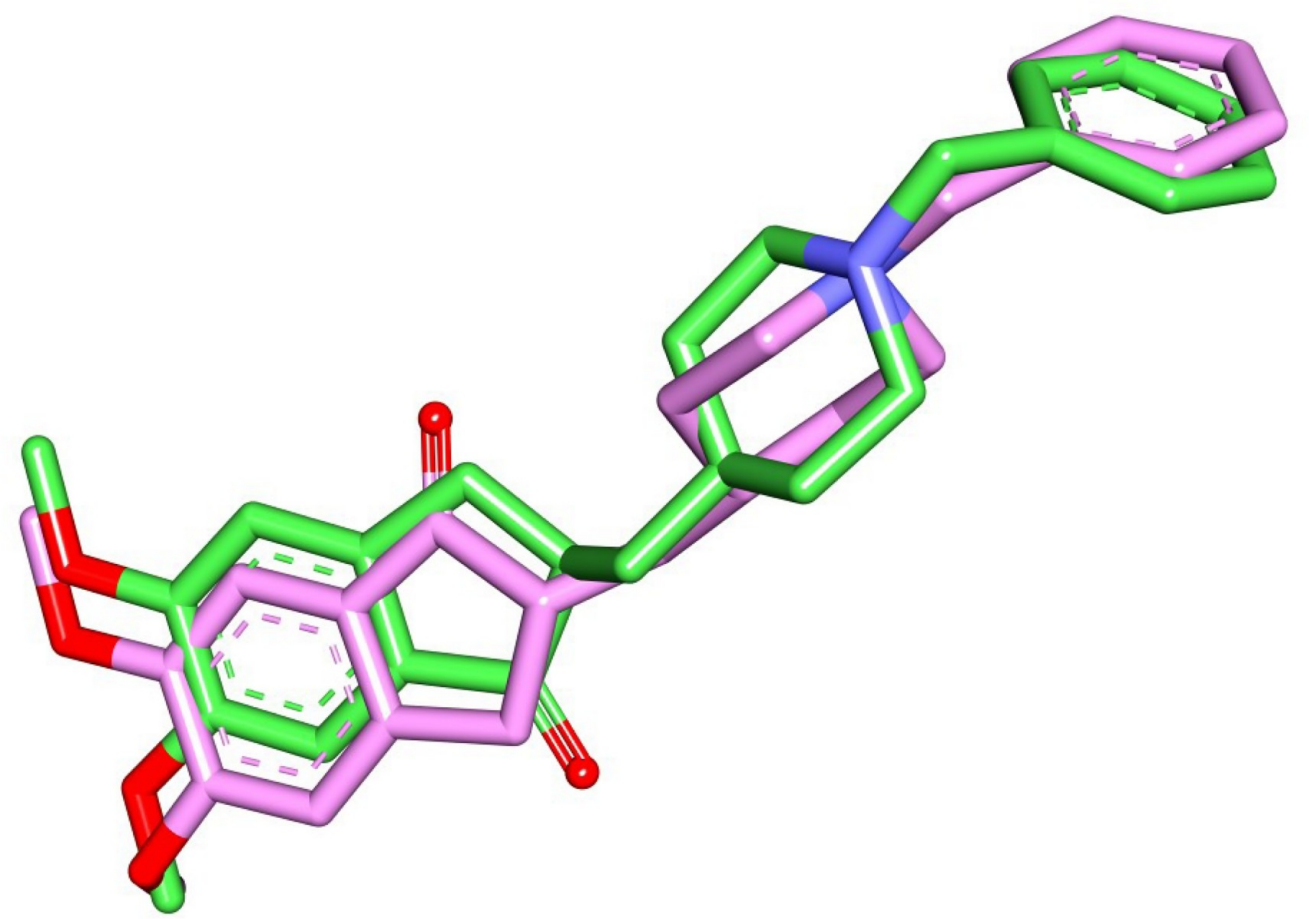
Predicted pose from molecular docking by Autodock Vina. Here, the stick representations of ligands denote the superimposed view of docked (pink) and co-crystallized ligand (green).

In order to check the binding modes of modified compounds, molecular docking simulations by Autodock Vina were performed. Molecular docking is one of the most common methods used in structure based drug design to analyze the interaction between a small molecule and a protein at the atomic level. Prior to docking, the crystal pose of donepezil was re-docked into the binding site of AChE with specific docking parameters and scoring functions, to check whether the docking software is reliable for the system. The conformation having the lowest negative score was then compared with the crystal pose. The value of the root mean square deviation (RMSD) of the docked conformation with respect to experimental conformation was 1.9659 Å (**Fig 4**), signifying the reliability of the docking protocol, as the threshold of reliability is 2.0 Å for a good docking protocol.

**Fig 5:**
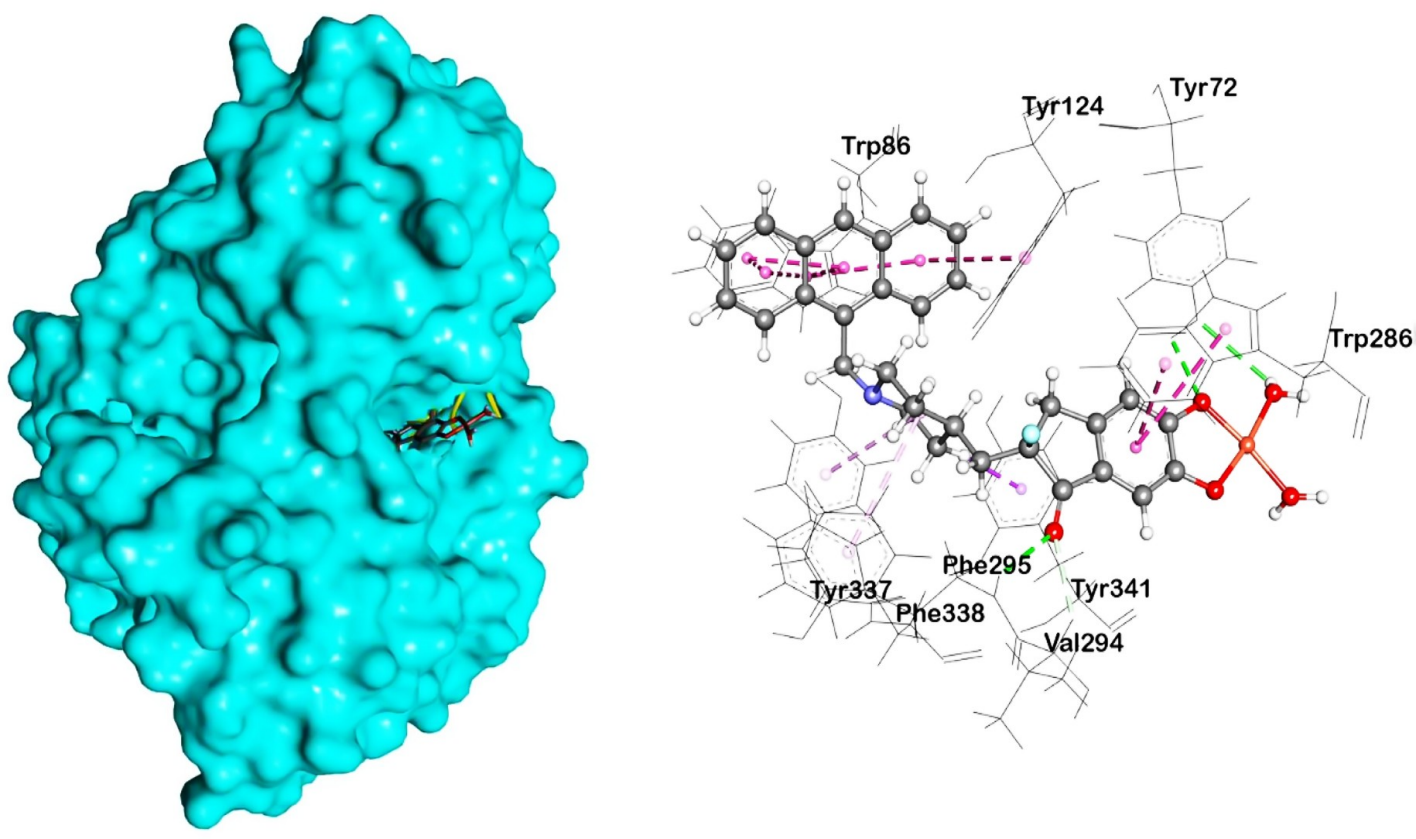
Predicted pose from the docking analysis showed the binding orientation map of important amino acids for analogue D9, showing hydrogen bond interaction (green color), including π–π stacking (pink color).

Afterward, all designed analogues were docked into the same binding site pocket of AChE, using similar optimized docking conditions. The outcomes of the docking analysis showed that all compounds, along with the parent compound, obtain binding affinities ranging from -10.2 to -14.9 kcal/mol. As shown in **Table 3**, D4, D5, D6 showed low binding affinities compared to parent compound, donepezil, while D1 exhibited high binding affinity. These results indicated that modification of Cu along with a water molecule increased the binding affinity, while addition of halogen groups like Br, I, and CF_3_ made some fluctuations in binding affinities; however, modification with naphthalene and anthracene rings increased the binding affinity. As shown with D7 and D8, obtained docking affinities of -13.9 and -14.8 kcal/mol were determined, respectively. The highest binding affinity was observed for the D9 compound. According to the post docking analysis, it was revealed that all compounds, except D6, showed π-alkyl interactions with Tyr337 and Phe338 residues of the PAS in the active site of the enzyme. D6 is positioned to form stabilizing π-alkyl interactions with Trp286, Tyr337, Tyr341 residues. Furthermore, it was also observed that modifications of donepezil increased the π-π interactions with the residues of the active site, while increasing their polarity resulted in the formation of hydrogen bonding interactions. The highest H-bonds were obtained for the D10 compound, forming with Gln291, Ser293, Phe295, Arg296 residues. In contrast, D7, D8, and D9 formed three H-bonds with Tyr72 and Phe295 residues. D8 and D9 showed similar binding conformations, despite having different bonding distances. Along with Trp286, D8, D9, and D10 displayed the maximum π- π interactions with the Trp86 residue denoting the tight binding with the activesite. Reports suggest that Trp286 is considered as the principal component of the PAS, responsible for the accessibility of small molecules to the active site and also in the allosterism, while aromatic interactions with the Trp286 residue modulates the inhibition constants for some AChE inhibitors [41,42]. As the D9 compound showed the highest binding affinity (**Fig 5**), it was subjected for subsequent MD, along with donepezil, to investigate the dynamic stability of the AChE-inhibitor complex, and also, to ensure the rationality of the sampling strategy.

**Table 3:** Binding affinity (kcal/mol) and nonbonding interactions of donepezil and its designed analogues.

Compound	Binding Affinity (kcal/mol)	Hydrophobic				Hydrogen Bond			
		Bonding Type	Protein	Ligand	Distance (Å)	Bonding Type	Protein	Ligand	Distance (Å)
			Interacting Amino Acids	Interacting Atoms or Rings			Interacting Amino Acids	Interacting Atoms or Rings	
Donepezil	-11.9	Pi-Alkyl	TYR337	X	5.344				
			PHE338	X	4.865				
			TYR341	X	5.342				
		Pi-Pi Stacked	TRP286	X1	4.175				
			TYR341	X1	5.303				
			TRP86	X	3.732				
			TRP86	X	4.373				
			TRP286	X1	5.466				
D1	-12.3	Pi-Alky	TYR337	X	5.084	Conventional	PHE295	H…O	1.917
			PHE338	X	5.194				
			TYR341	X	4.847				
		Pi-Pi Stacked	TRP286	X1	4.039				
			TYR341	X1	5.124				
			TRP86	X	3.731				
			TRP86	X	4.367				
			TRP286	X1	5.213				
D2	-12.7	Pi-Alkyl	TYR337	X	4.703	Conventional	TYR124	H…O	2.739
			PHE338	X	5.171				
			TYR341	X	4.613				
		Pi-Pi Stacked	TRP286	X1	4.068				
			TYR341	X1	5.302				
			TRP86	X	4.025				
			TRP86	X	4.430				
			TRP286	X1	5.355				
			HIS447	X	5.353				
		Pi-Sigma	TYR341	H…X	2.589				
D3	-12.4	Pi-Alkyl	TYR337	X	4.770	Conventional	PHE295	H…O	1.944
			PHE338	X	5.276				
			TYR341	X	4.497				
		Pi-Pi Stacked	TRP286	X1	4.017				
			TYR341	X1	5.387				
			TRP86	X	3.954				
			TRP86	X	4.301				
			TRP286	X1	5.244				
			HIS447	X	5.369				
D4	-11.2	Pi-Alkyl	TYR337	X	4.457	Conventional	PHE295	H…O	1.905
			PHE338	X	5.025				
			TYR341	X	4.665				
		Pi-Pi Stacked	TRP286	X1	4.202				
			TYR341	X1	5.099				
			TRP86	X	3.751				
			TRP86	X	4.341				
			TRP286	X1	5.541				
			HIS447	X	5.864				
D5	-10.2	Pi-Alkyl	TYR337	X	4.301	Conventional	PHE295	H…O	2.021
			PHE338	X	4.988				
			TYR341	X	4.797				
		Pi-Pi Stacked	TRP286	X1	4.367				
			TYR341	X1	5.015				
			TRP86	X	3.782				
			TRP86	X	4.410				
D6	-10.8	Pi-Alkyl	TRP286	X	4.113	Conventional	LEU289	H…O	1.922
			TYR337	X	4.138				
			TYR341	X	4.894				
		Pi-Pi T-Shaped	TYR337	X	5.922				
			TYR341	X	4.780				
D7	-13.9	Pi-Alkyl	TYR337	X2	5.408	Conventional	TYR72	C–H…O	2.717
			PHE338	X2	4.826				
							TYR72	C–H…O	2.745
							PHE295	C–H…O	2.497
		Pi-Pi Stacked	TRP286	X1	4.071				
			TRP86	X2	4.142				
			TRP86	X2	3.830				
			TRP86	X2	4.453				
			TRP86	X2	4.363				
			TRP286	X1	4.951				
		Pi-Sigma	TYR341	H…X2	2.735				
D8	-14.8	Pi-Alkyl	TYR337	X3	5.452	Conventional	TYR72	H…O	2.285
			PHE338	X3	4.739				
		Pi-Pi Stacked	TRP286	X1	4.119				
			TRP86	X3	4.501				
							TYR72	H…O	2.731
							PHE295	H…O	2.486
			TRP86	X3	3.884				
			TRP86	X3	4.154				
			TRP86	X3	4.683				
			TRP86	X3	4.977				
			TRP286	X1	5.003				
		Pi-Pi T-Shaped	TYR124	X3	5.784				
		Pi-Sigma	TYR341	H…X3	2.62				
D9	-14.9	Pi-Alkyl	TYR337	X3	5.367	Conventional	TYR72	H…O	3.069
							TYR72	H…O	2.741
							PHE295	H…O	2.755
			PHE338	X3	4.850				
		Pi-Pi Stacked	TRP286	X1	4.097				
			TRP86	X3	4.454				
			TRP86	X3	3.880				
			TRP86	X3	4.144				
			TRP86	X3	4.724				
			TRP86	X3	4.926				
			TRP286	X1	4.888				
		Pi-Pi T-Shaped	TYR124	X3	5.809				
		Pi-Sigma	TYR341	H…X3	2.48				
D10	-14.7	Pi-Alkyl	TYR337	X3	5.292	Conventional	GLN291	H…O	2.412
			PHE338	X3	5.289				
			TYR341	X3	3.914				
							SER293	H…O	2.380
		Pi-Pi Stacked	TRP286	X1	4.282		PHE295	H…O	1.667
							ARG296	H…O	2.851
			TRP86	X3	4.576				
			TRP86	X3	3.842				
			TRP86	X3	3.921				
			TRP86	X3	4.109				
			TRP86	X3	5.104				
			TRP286	X1	5.705				

Here X, X1, X2, X3 indicates that, X = Benzyl-4-piperidyl, X1 = 2,3-dihydro-1H-inden-1-one, X2 = Naphthalen-1-ylmethyl-4-piperidyl, X3 = Anthracen-9-ylmethyl-4-piperidyl

Furthermore, to understand how D9 showed its binding modes with different metals, different metal atoms such as Fe, Co, Zn and Ni were inserted to the same position of D9 where Cu is present and they are renamed as D9-Fe, D9-Co, D9-Zn and D9-Ni, respectively (**S1 Fig**). These analogues were optimized by DFT and the subsequent molecular docking was performed by the same protocol discussed above in the methods section. Afterward, obtained results were represented in **S1 Table**. As shown in **S1 Table**, D9-Fe, D9-Co, D9-Zn, D9-Ni showed low binding affinities compared to D9. As per the post docking analysis, it is shown that D9-Fe, D9-Co, D9-Zn showed the π-alkyl interactions with Tyr337 and Phe338 residues of the PAS of the active site of the enzyme, like D9, respectively, while the Val294 residue only formed π-alkyl interaction with D9-Ni. In addition, it was revealed that all of the modified D9 compounds, except D9-Ni, showed maximum π-π interactions with Trp286 and Trp86. D9-Ni formed major π-π interaction with Trp286 along with the Tyr341 residue, which was also observed in D9-Co and D9-Zn. Furthermore, D9-Co and D9-Zn formed a hydrogen bond with the Phe295 residue while D9-Fe forms H-bonding with both Phe295 and Tyr72 residue, as like the D9 compound (illustrated in **S2 Fig**). From the different metal based study of D9, analysis finally revealed that D9-Cu performs better binding than other candidates.

### Molecular dynamics simulations

**Fig 6:**
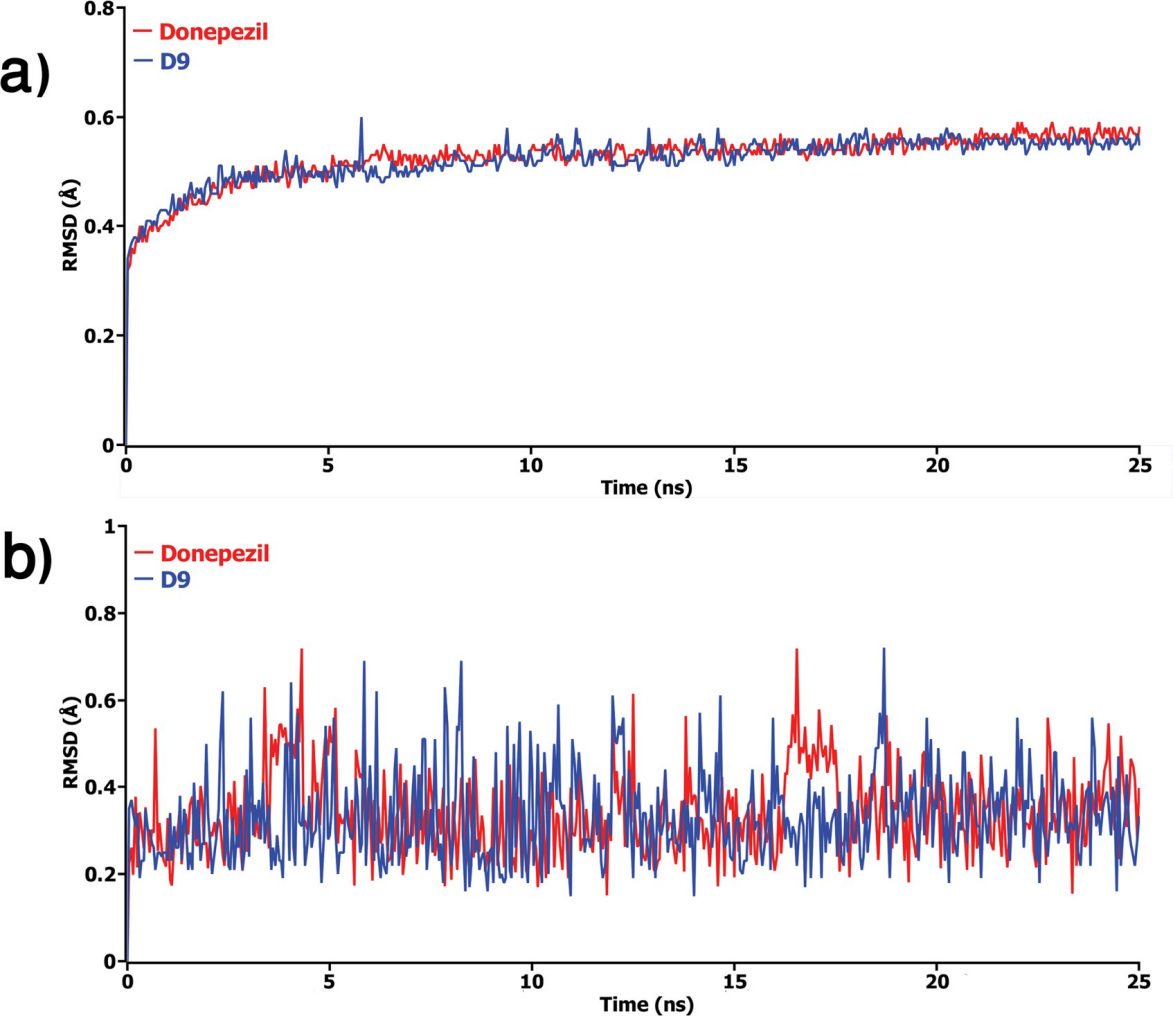
The time series of the RMSD of backbone atoms (C, Cα, and N) for a) protein and b) ligand for each docked complex. Here, red and blue lines denote donepezil and D9 complex respectively.

In order to understand the binding mechanism, structural behavior, and flexibility of compound D9, we performed MD simulations for 25 ns. The complex of donepezil-protein was also subjected to MD simulation as a reference compound. The atomic RMSDs of the Cα atoms for both protein and the ligand of each complex were calculated and plotted in a time dependent manner (**Fig 6)**. **Fig 6A** demonstrats the behavior of the protein during the simulation; in which, both complexes were observed to achieve equilibrium after 5 ns and fluctuated around 0.5 Å. However, after 20 ns, D9 complex showed lower RMSD and remained afterward. Similar results were also obtained for ligands of each complex, as shown in **Fig 6B**. As can be seen in the plot, high fluctuation in RMSDs was observed for donepezil, where the high magnitudes were observed at 16 ns to 18 ns. The RMSD results from both protein and ligand indicated that the complexes were stable, suggesting higher stability of D9 in comparison with donepezil.

**Fig 7:**
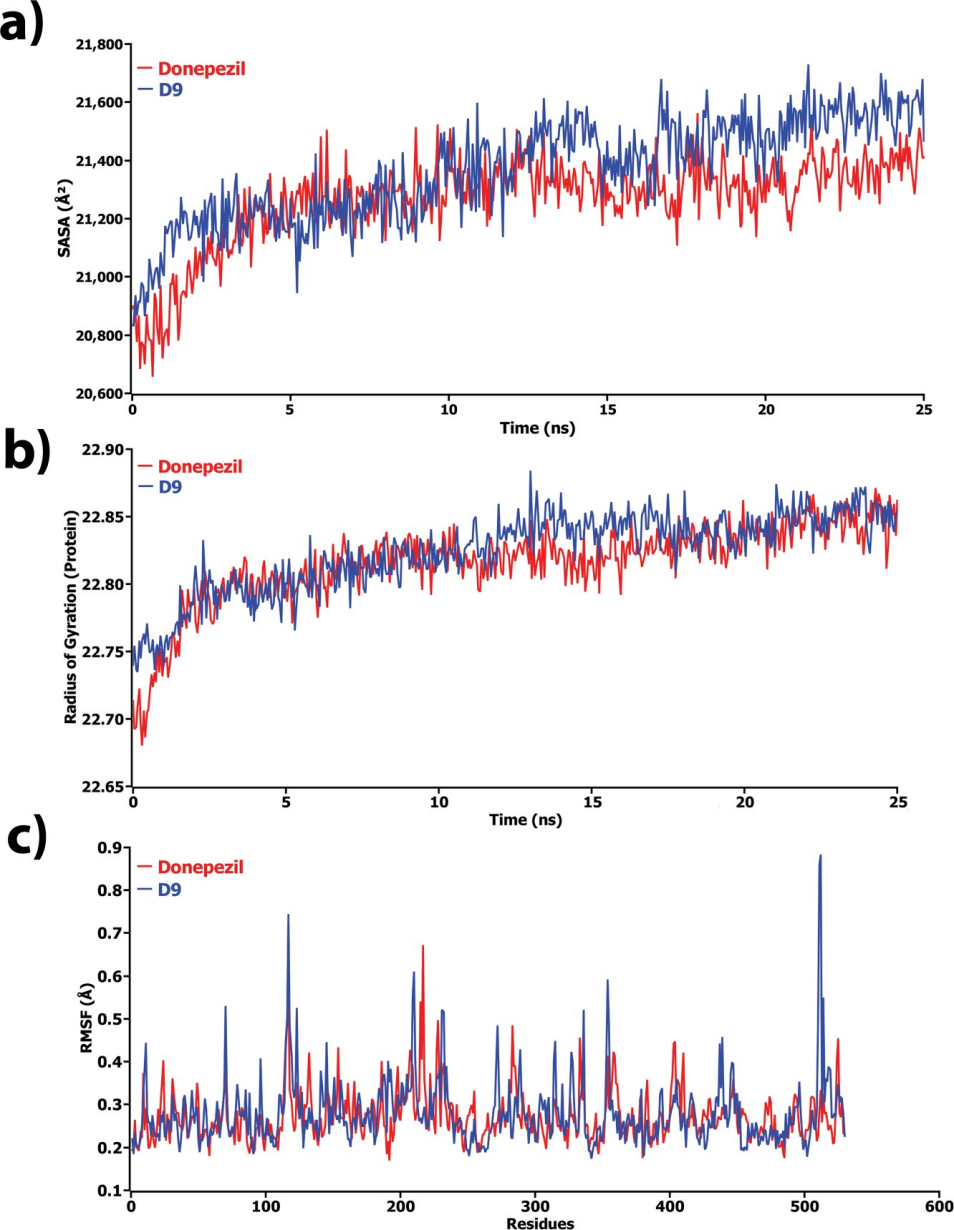
The structural changes of protein by means of a) solvent accessible surface area (SASA), b) radius of gyration, and c) root means square fluctuations (RMSF) analysis. Here, red and blue lines denote donepezil and D9 complex, respectively.

**Fig 8:**
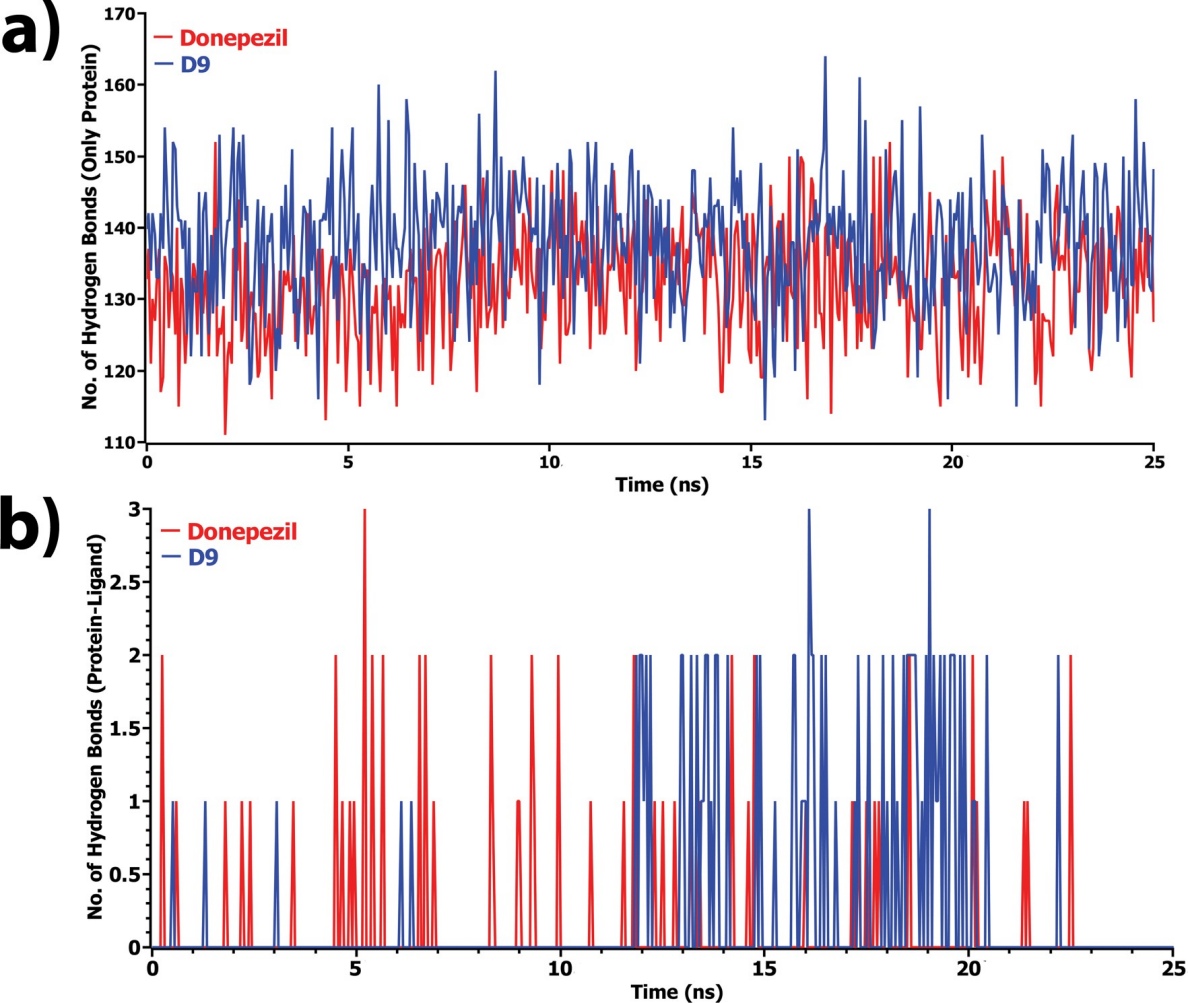
Total number of hydrogen bonds formed a) within the protein and b) between the protein and ligand in complex state during the simulation. Here, red and blue lines denote donepezil and D9 complex, respectively.

**Fig 9:**
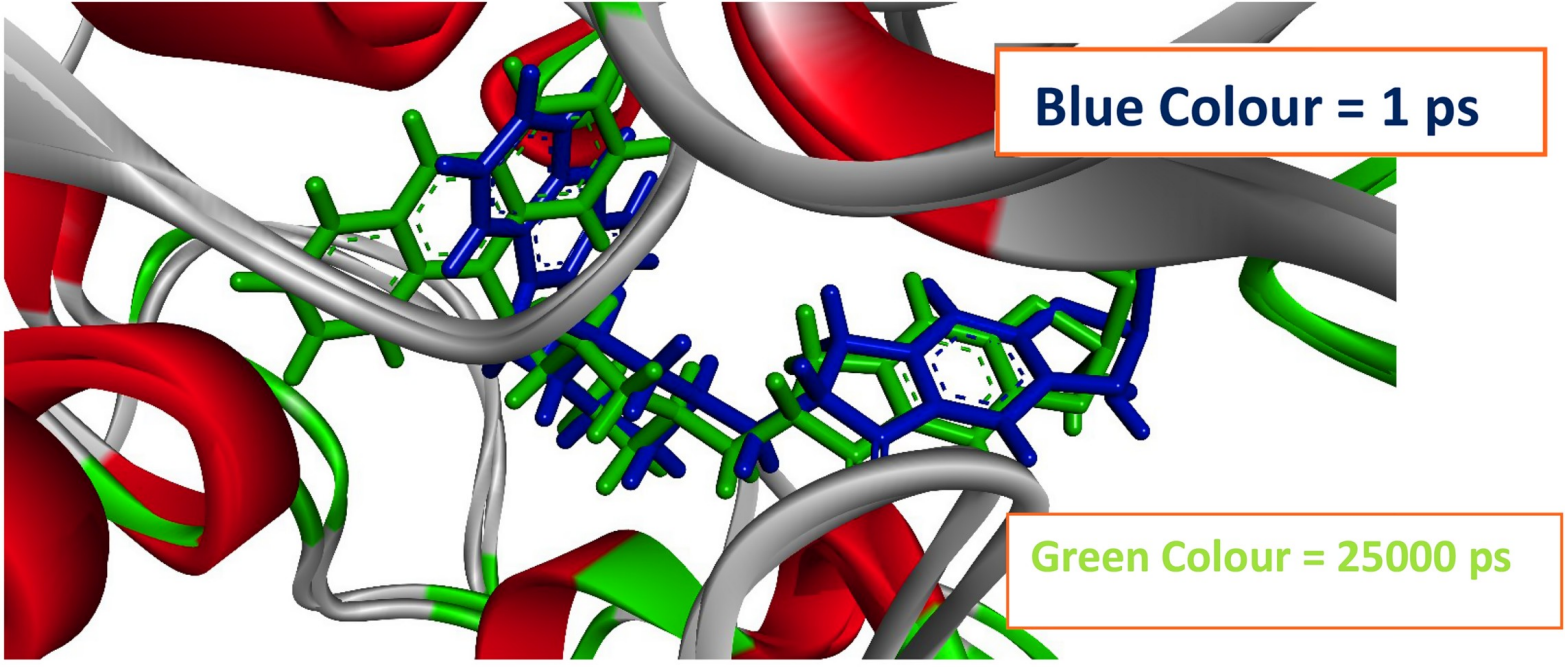
Conformational changes of the D9-AChE complex. Here, the stick model of the ligand in blue color represents the starting conformation of the complex, while the green color represents the conformation of last step in the 25 ns long MD simulation.

For better understanding on how D9 and donepezil influence the binding mode with AChE, the structural changes of two complexes were examined by means of root mean square fluctuation (RMSF), radius of gyration, and solvent accessible surface area (SASA) of the protein (**Fig 7**). **Fig 7A** represents the total SASA of each protein, in which the D9 compound showed decreased SASA after 15 ns of simulation, demonstrating lower compactness of the protein structure. In contrast, the results from the radius of gyration analysis (**Fig 7B)** described that D9 comparatively produced a higher radius of gyration value than donepezil, denoting loose packing of the protein structure, which eventfully supported the results from the SASA analysis. RMSF values were also calculated from the trajectories, which reflect the flexibility of each residue in the protein[43][43][43][43][43][43][43][43][43][42][41][40][40][40][40][40][39]. According to **Fig 7C**, it was observed that D9 induced flexibility to some residues in the protein. Highest fluctuations were observed in several regions, ranging from 116–125, 280–290, 310–320, 361–370, and 505–515. Finally, the information of hydrogen bonding interactions formed within the protein, and also between the protein and ligand at the catalytic domain, was collected from the trajectories and represented in **Fig 8A**. Here, the D9 complex showed maximum intramolecular hydrogen bonds in the donepezil complex, demonstrating the stability of the complex. The intermolecular hydrogen bond analysis between the protein and ligand displayed that donepezil and D9 formed hydrogen bonds with the residues of the catalytic domain (**Fig 8B**). At the initial step, D9 did not show much interaction; however, after 11 ns, it showed several H-bond contacts. Consequently, donepezil revealed no hydrogen bonding in the docking pose, although it detected several contacts during the simulations. As a corollary, all analyses from the MD simulations suggested that D9 is more stable than donepezil and caused little conformational changes of the protein by undergoing little movement during the MD simulations **(Fig 9)**.

### Ensemble based docking

**Fig 10:**
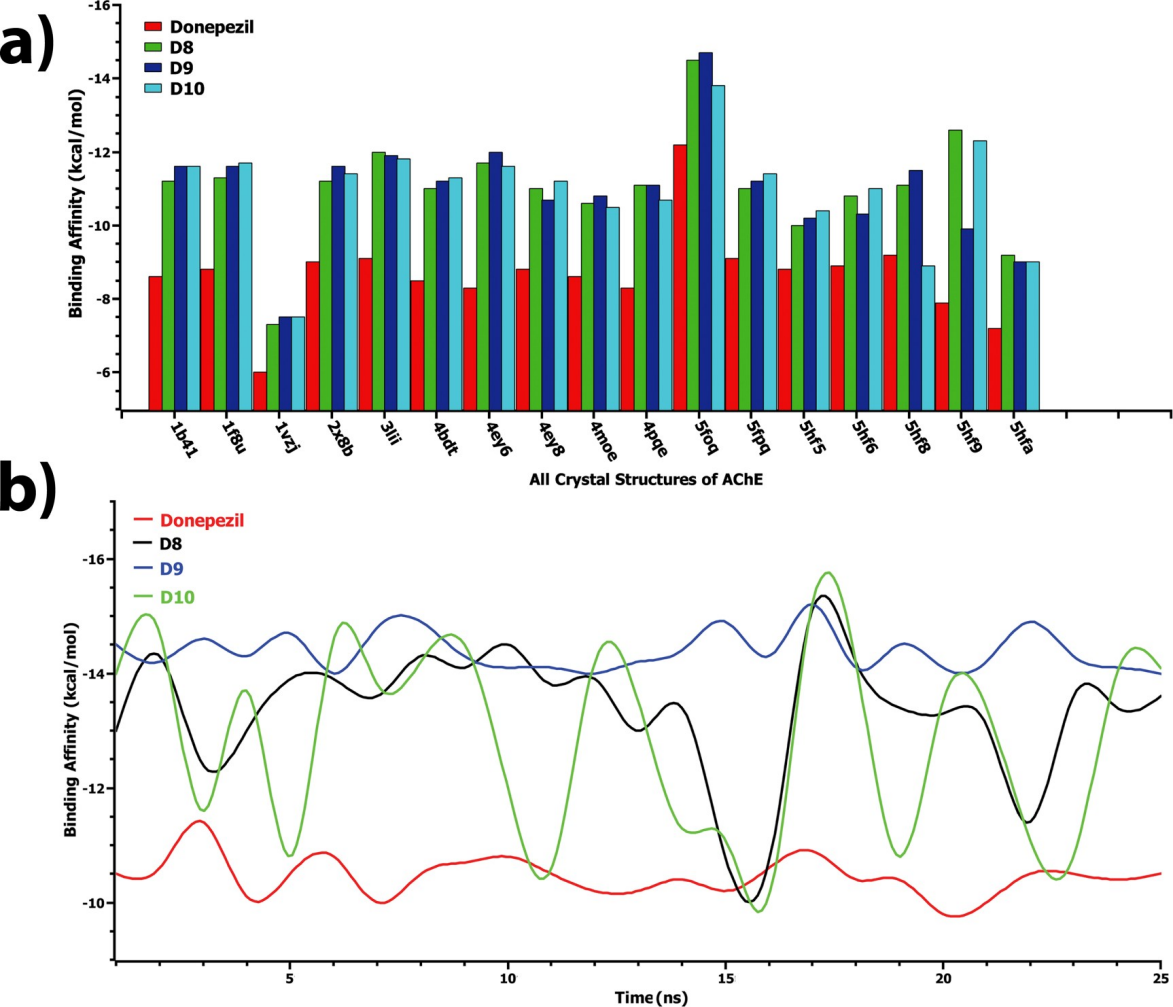
Binding affinities of designed ligands and standard inhibitor, obtained from ensemble based docking analysis. Here, binding affinities of D8, D9, D10, and donepezil against multiple AChE conformers from a) protein data bank and b) 25 ns MD simulation.

Usually, proteins are flexible macromolecules in nature. However, this property significantly influences ligand binding, especially in molecular reorganization and interactions [44]. Compared to the other program, AutoDock Vina is the most popular docking program to determine the binding pose of the ligand, yet suffers from backbone flexibility in receptors. Therefore, ensemble based molecular docking by AutoDock Vina has been introduced in this study to overcome this limitation. The results obtained are represented in **Table 4 and Table 5**. **Table 4** and **Fig 10A** describe the binding affinity of all ligands with different crystallographic conformations of the AChE enzyme. Interestingly, designed compounds showed better results than the standard drug, donepezil. Among these crystal conformers, designed compounds and donepezil produced best docking scores against the 5foq conformer (**Fig 10A)**, and therefore, detailed molecular interactions of this conformer have been investigated and illustrated in **Table 6**. As can be seen in **Table 6**, the D9 compound formed two additional hydrophobic interactions with Tyr341 and Trp286 residues followed by π-alkyl and π-π stacked bonds, and also obtained the highest docking score. In case of D8 compound, additional hydrogen bonds were observed with Ser293 and Trp286 residues, while the polar interactions with Tyr72 and Phe295 were seen to disappear. Similarly, loss of hydrogen bonds was also observed for Gln291, Phe295, and Arg296 residues. All ligands showed better binding affinities against the conformer obtained from MD simulation at 17 ns, as shown in **Table 5** and **Fig 10B**. Detailed molecular interactions were illustrated in **Table 6** which represents the breakdown of π-alkyl interactions of Tyr341, Tyr337, and Tyr124 residues. Instead, they formed π-π stacking with the ligands. Also, D9 and D10 compounds showed an additional salt bridge with the Asp74 residue followed by π-cation interactions. It is noteworthy to state that the flexibility of AChE is the major determinant of the binding affinity of the ligands, as evident from ensemble-based docking. π-π stacking with the residues of Trp86, Tyr337, Tyr341, Tyr124, and Trp286 showed a major contribution for strong drug binding and activity. Our study suggested that protein flexibility can give rise to differences in binding affinity and binding interaction of a drug with its target protein.

**Table 4:** Ensemble based docking against all crystal structures of AChE.

PDB ID	Resolution (Å)	Sequence Positions	Donepezil	D8	D9	D10
1b41	2.76	36–574	-8.6	-11.2	-11.6	-11.6
1f8u	2.90	32–614	-8.8	-11.3	-11.6	-11.7
1vzj	2.35	575–614	-6	-7.3	-7.5	-7.5
2x8b	2.95	32–614	-9	-11.2	-11.6	-11.4
3lii	3.20	35–574	-9.1	-12	-11.9	-11.8
4bdt	3.10	32–614	-8.5	-11	-11.2	-11.3
4ey6	2.40	33–574	-8.3	-11.7	-12	-11.6
4ey8	2.60	33–574	-8.8	-11	-10.7	-11.2
4moe	2.00	33–574	-8.6	-10.6	-10.8	-10.5
4pqe	2.90	32–574	-8.3	-11.1	-11.1	-10.7
5foq	2.30	32–576	-12.2	-14.5	-14.7	-13.8
5fpq	2.40	33–574	-9.1	-11	-11.2	-11.4
5hf5	2.15	33–574	-8.8	-10	-10.2	-10.4
5hf6	2.30	33–574	-8.9	-10.8	-10.3	-11
5hf8	2.80	33–574	-9.2	-11.1	-11.5	-8.9
5hf9	2.20	33–574	-7.9	-12.6	-9.9	-12.3
5hfa	2.20	33–574	-7.2	-9.2	-9	-9

**Table 5:** Binding affinity values of donepezil, D8, D9, and D10 docked against multiple AChE conformers generated by 25 ns MD simulation.

MD Conformers	Donepezil	D8	D9	D10
1ns	-10.5	-13.0	-14.5	-14.0
2ns	-10.6	-14.3	-14.2	-14.7
3ns	-11.4	-12.4	-14.6	-11.6
4ns	-10.1	-13	-14.3	-13.7
5ns	-10.5	-13.9	-14.7	-10.8
6ns	-10.8	-13.9	-14.0	-14.6
7ns	-10.0	-13.6	-14.8	-13.8
8ns	-10.5	-14.3	-14.9	-14.2
9ns	-10.7	-14.1	-14.3	-14.5
10ns	-10.8	-14.5	-14.1	-12.0
11ns	-10.5	-13.8	-14.1	-10.6
12ns	-10.2	-13.9	-14.0	-14.2
13ns	-10.2	-13.0	-14.2	-13.5
14ns	-10.4	-13.4	-14.4	-11.3
15ns	-10.2	-10.8	-14.9	-11.1
16ns	-10.6	-10.7	-14.3	-10.1
17ns	-10.9	-15.1	-15.2	-15.2
18ns	-10.4	-14.2	-14.1	-14.2
19ns	-10.4	-13.4	-14.5	-10.8
20ns	-9.8	-13.3	-14.1	-13.5
21ns	-10.0	-13.1	-14.2	-13.4
22ns	-10.5	-11.4	-14.9	-11.1
23ns	-10.5	-13.6	-14.3	-10.8
24ns	-10.4	-13.4	-14.1	-14.0
25ns	-10.5	-13.6	-14	-14.1

**Table 6:** Nonbonding interactions of the best docked complexes obtained from ensemble based docking analysis.

Conformers	Compounds	Hydrophobic				Hydrogen Bond				Electrostatic			
		Bonding Type	Protein	Ligand	Dista nce (Å)	Bonding Type	Protein	Ligand	Distan ce (Å)	Bondi ng Type	Protein	Ligand	Distance (Å)
			Interacting Amino Acids	Interacting Atoms or Rings			Interacting Amino Acids	Interacting Atoms or Rings			Interacting Amino Acids	Interacting Atoms or Rings	
5foq	Donepezil	Pi-Alkyl	TYR337	X	5.074	Conventional	PHE295	H…O	1.944				
			TYR341	X	4.964								
		Pi-Pi Stacked	TRP86	X1	3.844								
			TRP86	X1	3.851								
			TRP286	X	5.086								
			TRP286	X	3.890								
			TYR341	X1	5.605								
		Pi-Pi T-Shaped	TYR337	X	5.314								
		Pi-Sigma	TRP286	H…X	3.689								
	D8	Pi-Alkyl	TYR337	X2	5.338	Conventional	SER293	H…O	3.362				
			PHE338	X2	5.069								
			TYR341	X2	5.108								
		Pi-Pi Stacked	TRP86	X2	4.661	Pi-Donor	TRP286	H… π	4.121				
			TRP86	X2	5.402								
			TRP86	X2	4.397								
			TRP86	X2	4.055								
			TRP86	X2	4.884								
			TRP286	X1	5.227								
			TRP286	X1	4.048								
		Pi-Pi T-Shaped	TYR337	X2	5.314								
	D9	Pi-Alkyl	TYR337	X2	5.421	Conventional	TYR124	H…O	2.989				
			PHE338	X2	5.032								
			TYR341	X2	5.224								
		Pi-Pi Stacked	TRP86	X2	4.651								
			TRP86	X2	5.426								
			TRP86	X2	4.405								
			TRP86	X2	4.041								
			TRP86	X2	4.885								
			TRP286	X1	5.233								
			TRP286	X1	3.987								
	D10	Pi-Alkyl	TYR337	X2	5.486	Conventional	TYR124	H…O	3.086				
			PHE338	X2	4.986								
							SER293	H…O	3.375				
			TYR341	X2	5.311								
		Pi-Pi Stacked	TRP86	X2	4.663								
			TRP86	X2	5.419								
			TRP86	X2	4.409								
			TRP86	X2	4.052								
			TRP86	X2	4.888								
			TRP286	X1	5.260								
			TRP286	X1	3.951								
17ns	Donepezil	Pi-Alkyl	PHE338	X	4.624	Conventional	TYR124	H…O	2.332				
		Pi-Pi Stacked	TRP86	X	5.655								
			TRP86	X	5.717								
			TRP286	X1	5.690								
			TRP286	X1	4.545								
			TYR337	X	4.055								
			TYR341	X1	4.479								
	D8	Pi-Alkyl	PHE338	X2	4.992	Conventional	TYR124	H…O	2.251	Pi-Anion	ASP74	O…X2	4.64
							TYR72	H…O	2.026				
							TYR72	H…O	3.065				
		Pi-Pi T-Shaped	TRP86	X2	3.738	Pi-Donor	TRP286	H…π	3.879				
			TRP86	X2	5.618								
			TRP86	X2	4.092								
			TRP86	X2	4.524								
			TRP86	X2	5.860								
			TRP86	X2	4.608								
			TRP286	X1	5.163								
			TRP286	X1	4.244								
			TYR337	X2	3.981								
			TYR337	X2	5.075								
			TYR341	X1	4.700								
	D9	Pi-Alkyl	TYR124	X2	5.476	Conventional	TYR124	H…O	2.539	Pi-Anion	ASP74	O…X2	4.467
			PHE338	X2	5.257								
		Pi-Pi Stacked	TRP86	X2	3.713								
			TRP86	X2	5.660								
			TRP86	X2	4.109								
			TRP86	X2	4.343								
			TRP86	X2	5.805								
			TRP86	X2	4.484								
			TRP286	X1	5.144								
			TRP286	X1	4.408								
			TYR337	X2	3.991								
			TYR337	X2	5.086								
			TYR341	X1	4.591								
	D10	Pi-Alkyl	PHE338	X2	5.208	Conventional	PHE295	C–H…O	2.882	Pi-Anion	ASP74	O…X2	4.500
							SER293	C–H…O	2.839				
							SER293	C–H…O	3.323				
			VAL294	X2	5.317								
		Pi-Pi Stacked	TRP86	X2	3.869								
			TRP86	X2	5.260								
			TRP86	X2	3.912								
			TRP86	X2	4.645								
			TRP86	X2	5.466								
			TRP86	X2	4.424								
			TYR337	X1	4.165								
			TYR337	X1	5.382								
		Pi-Sigma	TYR124	H…X2	3.391								

Here X, X1, X2 indicates that, X = Benzyl-4-piperidyl, X1 = 2,3-dihydro-1H-inden-1-one, X2 = Anthracen-9-ylmethyl-4-piperidyl

### ADME/T analysis

In order to analyze whether the modified compounds produce any toxicity or altered pharmacokinetic profile, the admetSAR server was utilized. Different pharmacokinetic and pharmacodynamic parameters such as human intestinal absorption, [45] blood–brain barrier penetration, cytochrome P450 inhibition, [46] human ether-a-go-go-related genes inhibition, acute oral Toxicity, and rat acute toxicity were considered. The results are summarized in **Table 7**. As shown in **Table 7**, all compounds revealed a positive value (value above the prescribed threshold suggesting good permeability) with high probabilities, in case of blood-brain barrier and human intestinal absorption. Furthermore, modifications of donepezil resulted in a non–inhibitor of P-glycoprotein. The analysis displayed that D2, D4, D5, D6 and D9 were potential compounds of the human ether-a-go-go-related gene. All compounds showed a similar oral toxicity profile, while D9 and D2 indicated the highest LD_50_ value in rat acute toxicity, demonstrating non-toxic with respect to parent donepezil.

**Table 7:** Selected pharmacokinetic parameters of donepezil and its designed analogues.

Parameters	Donepezil	D1	D2	D3	D4	D5	D6	D7	D8	D9	D10
Blood-Brain Barrier	+	+	+	+	+	+	+	+	+	+	+
	(0.99)	(0.93)	(0.93)	(0.90)	(0.90)	(0.89)	(0.91)	(0.93)	(0.93)	(0.93)	(0.90)
Human Intestinal Absorption	+	+	+	+	+	+	+	+	+	+	+
	(0.99)	(0.66)	(0.79)	(0.79)	(0.72)	(0.58)	(0.60)	(0.66)	(0.66)	(0.79)	(0.79)
P-glycoprotein Inhibitor	I	NI	NI	NI	NI	NI	NI	NI	NI	NI	NI
	(0.69)	(0.90)	(0.83)	(0.82)	(0.76)	(0.84)	(0.85)	(0.90)	(0.90)	(0.83)	(0.82)
CYP450 2C9 Inhibitor	NI	NI	NI	NI	NI	NI	NI	NI	NI	NI	NI
	(0.88)	(0.83)	(0.80)	(0.80)	(0.79)	(0.79)	(0.78)	(0.83)	(0.83)	(0.80)	(0.80)
Human Ether-a-go-go-Related Gene	I	NI	I	NI	I	I	I	NI	NI	I	NI
	(0.68)	(0.61)	(0.58)	(0.50)	(0.51)	(0.50)	(0.56)	(0.61)	(0.61)	(0.58)	(0.50)
Acute Oral Toxicity	III	III	III	III	III	III	III	III	III	III	III
	(0.56)	(0.55)	(0.55)	(0.54)	(0.54)	(0.54)	(0.55)	(0.55)	(0.55)	(0.55)	(0.54)
Rat Acute Toxicity (LD50, mol/kg)	2.70	2.59	2.72	2.71	2.69	2.69	2.71	2.59	2.59	2.72	2.71

+ = Positive, I = Inhibitor, NI = Non-Inhibitor, III = Category III includes compounds with LD50 values greater than 500mg/kg but less than 5000mg/kg.

In addition, ADME/T prediction of D9-Fe, D9-Co, D9-Zn, and D9-Ni was performed and compared with the D9-Cu analogue. D9 with different metals revealed the same values as D9-Cu. An exception, D9-Zn, showed a negative value in human oral absorption. These results have been summarized in S2 Table. A published review by Mjos *et al*. 2014 [47] discussed the importance of metal based drugs in the diagnosis of disease and enlisted a number of metal-based drug names which are already approved by the FDA and which have undergone clinical trials (shown in **S3 Table**). From **S3 Table,** we also predicted the pharmacokinetic parameters and toxicity of some drugs (data is shown in **S4 Table**).

## Conclusion

In summary, the present study revealed some novel metal directed AChE inhibitors, developed by modifying a known inhibitor, donepezil. Modification with Cu, along with substitution using aromatic rings and halogens increased the dipole moment and π-π interaction capacity of the designed compounds. Furthermore, these modified compounds were more reactive than donepezil, as they showed lower HOMO‒LUMO gaps. Molecular interaction analyses of docking simulations revealed similar binding conformations of all compounds at the active site and suggested D9 as a potent inhibitor, which can equally interact with both the CAS (Trp86) and PAS sites (Trp286) of AChE. The structural analysis with subsequent MD simulations demonstrated that D9 formed a stable conformation by creating hydrophobic and aromatic interactions with the active site residues such as Tyr337, Phe295, Tyr72, and Phe338. In addition, π-π stacking interactions with the residues of Trp86, Tyr337, Tyr341, Tyr124, and Trp286 may play a major role for strong drug binding and activity, according to ensemble based docking. Moreover, ADME/T analyses suggested that modified analogues were less toxic and have improved pharmacokinetic profiles than the parent drug. These results further confirmed the ability of Cu and other metal-directed analogues to bind simultaneously to the active sites of AChE and support them as potential candidates for the future treatment of Alzheimer’s disease.

## Supporting information

S1 Table Binding affinity and nonbonding interaction of D9 including different metal form. (DOCX)

S2 Table Selected pharmacokinetic parameter of D9-Fe, D9-Co, D9-Zn & D9 Ni. (DOCX)

S3 Table List of important and promising metal drugs (Chem. Rev. 2014, *114 (8)*, 4540–4563). (DOCX)

S4 Table Selected pharmacokinetic parameter of some metal based FDA approved, clinical trials and promising drugs. (DOCX)

S1 Fig Most stable optimized structures of D9-Fe, D9-Co, D9-Zn and D9-Ni along with D9. (TIF)

S2 Fig Predicted pose from docking analysis showed the binding orientation map of important amino acids for **a) D9-Fe, b) D9-Co, c) D9-Zn and d) D9-Ni**, showing hydrogen bond interaction (green color), including π–π stacking (pink color).
(TIF)
