# Supplementary material for: Metal based donepezil analogues designed to inhibit human acetylcholinesterase for Alzheimer’s disease
Source: PLoS One. 2019 Feb 20;14(2):e0211935. doi: 10.1371/journal.pone.0211935 (PMC6382135; doi:10.1371/journal.pone.0211935)
Supplement: S1 Table — (DOCX) [file pone.0211935.s001.docx]

**S1 Table.** Binding affinity and nonbonding interaction of D9 including different metal form

| **Compound** | **Binding Affinity**  **(kcal/mol)** | **Hydrophobic** | | | | **Conventional Hydrogen Bond** |
| --- | --- | --- | --- | --- | --- | --- |
|  |  | **Pi-Alkyl** | **Pi-Pi Stacked** | **Pi-Pi T- Shaped** | **Pi-Sigma** |  |
| **D9-Fe** | -14.7 | TYR337  (5.213)  PHE338  (4.871) | TRP86  (4.172)  TRP86  (4.728)  TRP86  (4.968)  TRP86  (4.494)  TRP86  (3.896)  TRP286  (4.921)  TRP286  (4.128) | TYR124  (5.779) | TYR341  (2.539) | PHE295  (2.734)  TYR72  (2.457) |
| **D9-Co** | -14.4 | PHE338  (4.887)  TYR337  (4.775) | TRP86  (4.218)  TRP86  (5.046)  TRP86  (4.768)  TRP86  (3.975)  TRP86  (4.567)  TRP286  (4.962)  TRP286  (3.980)  TYR341  (5.171) | TYR124  (5.742) | TYR337  (2.671) | PHE295  (2.211) |
| **D9-Zn** | -14.3 | TYR337  (5.006)  PHE338  (5.027)  TYR341  (4.767) | TRP86  (4.199)  TRP86  (5.046)  TRP86  (4.736)  TRP86  (4.540)  TRP86  (3.942)  TRP286  (5.033)  TRP286  (3.999)  TYR341  (5.243) | TYR124  (5.768) |  | PHE295  (2.046) |
| **D9-Ni** | -10.6 | VAL294  (5.493) | TRP286  (3.829)  TRP286  (4.050)  TRP286  (4.986)  TRP286  (4.796)  TRP286  (3.772)  TYR341  (5.363)  TYR341  (4.638) |  |  | GLN291  (2.416) |
