# Supplementary material for: Metal based donepezil analogues designed to inhibit human acetylcholinesterase for Alzheimer’s disease
Source: PLoS One. 2019 Feb 20;14(2):e0211935. doi: 10.1371/journal.pone.0211935 (PMC6382135; doi:10.1371/journal.pone.0211935)
Supplement: S2 Table — (DOCX) [file pone.0211935.s002.docx]

**S2 Table**. Selected pharmacokinetic parameter of D9-Fe, D9-Co, D9-Zn & D9 Ni

| **Parameters** | **Blood**  **-Brain**  **Barrier** | **Human**  **Intestinal**  **Absorption** | **P-glycoprotein**  **Inhibitor** | **CYP450 2C9**  **Inhibitor** | **Human**  **Ether-a-**  **go-go-**  **Related**  **Gene** | **Acute Oral**  **Toxicity** | **Rat**  **Acute**  **Toxicity**  **(LD50,mol/k**  **g)** |
| --- | --- | --- | --- | --- | --- | --- | --- |
| **Compounds** |  |  |  |  |  |  |  |
| **D9-Fe** | +  (0.93) | +  (0.88) | NI  (0.80) | NI  (0.78) | I  (0.65) | iii  (0.56) | 2.74 |
| **D9-Co** | +  (0.93) | +  (0.88) | NI  (0.80) | NI  (0.78) | I  (0.65) | iii  (0.56) | 2.74 |
| **D9-Zn** | +  (0.92) | -  (0.61) | NI  (0.80) | NI  (0.68) | I  (0.64) | iii  (0.56) | 2.73 |
| **D9-Ni** | +  (0.93) | +  (0.88) | NI  (0.80) | NI  (0.78) | I  (0.65) | iii  (0.56) | 2.74 |

+ =Positive, - = Negative I= Inhibitor, NI =Non-Inhibitor, III = Category III includes compounds with LD50 values greater than 500mg/kg but less than 5000mg/kg.
