# Supplementary material for: Metal based donepezil analogues designed to inhibit human acetylcholinesterase for Alzheimer’s disease
Source: PLoS One. 2019 Feb 20;14(2):e0211935. doi: 10.1371/journal.pone.0211935 (PMC6382135; doi:10.1371/journal.pone.0211935)
Supplement: S3 Table — (DOCX) [file pone.0211935.s003.docx]

**S3 Table.** List of important and promising metal drugs (Chem. Rev. **2014,** *114 (8)*, 4540-4563).

| **Sl No** | **Metal Based Drug List** | **Used in Disease** |
| --- | --- | --- |
| **A.** | **FDA Approved** |  |
|  |  |  |
| 1 | Cisplatin | Cancer therapy |
| 2 | Carboplatin | Cancer therapy |
| 3 | Oxaliplatin | Cancer therapy |
| 4 | Porfilmer Sodium | Photodynamic therapy |
| 5 | Melarsoprol | Antimicrobial and antiparasitic |
| 6 | Meglumine antimoniate | Antimicrobial and antiparasitic |
| 7 | Sodium stibugluconate | Antimicrobial and antiparasitic |
| 8 | CBS | Antimicrobial and antiparasitic |
| 9 | Silver sulphadiazine | Antimicrobial and antiparasitic |
| 10 | Xeroform | Antimicrobial and antiparasitic |
| 11 | Sodium aurothiomalate | Antimicrobial and antiparasitic |
| 12 | Aurothioglucose | Antimicrobial and antiparasitic |
| 13 | Sodium aurothiopropanolsulfonate | Antimicrobial and antiparasitic |
| 14 | Sodium aurothiosulfate | Antimicrobial and antiparasitic |
| 15 | Auranofin | Antimicrobial and antiparasitic |
| 16 | AMD3100 | Metal chelators for cancer therapy |
| 17 | Vorinostat | Metal chelators for cancer therapy |
|  |  |  |
| **B.** | **Clinical Trials** |  |
|  |  |  |
| 18 | Nedaplatin | Cancer therapy |
| 19 | Lobaplatin | Cancer therapy |
| 20 | Heptaplatin | Cancer therapy |
| 21 | Satraplatin | Cancer therapy |
| 22 | NAMI-A | Cancer therapy |
| 23 | KP1019 | Cancer therapy |
| 24 | (N)KP1339 | Cancer therapy |
| 25 | Darinaparsin | Cancer therapy |
| 26 | Padeliporfin | Photodynamic therapy |
| 27 | Ferroquine | Antimicrobial and antiparasitic |
| 28 | Clloquinol | Metal chelators in clinical trials for the treatment of neurodegenerative diseases. |
| 29 | PBT2 | Metal chelators in clinical trials for the treatment of neurodegenerative diseases. |
| 30 | PXD101 | Metal chelators for cancer therapy |
| 31 | Givinostat | Metal chelators for cancer therapy |
|  |  |  |
| **C.** | **Promising Future Candidates** |  |
|  |  |  |
| 32 | BMOV | Diabetes |
| 33 | BEOV | Diabetes |
| 34 | CTC-96 | Antiviral |
| 35 | Sodium Thiomersal | Antiviral |
| 36 | M40403 | Cardiovascular disorders |
| 37 | BSS | Gastrointestinal disorders |
| 38 | RM175 | Organometallic ruthenium(II) complexes with promising anticancer activity |
| 39 | RAPTA-C | Organometallic ruthenium(II) complexes with promising anticancer activity |
| 40 | NP309 | Organometallic ruthenium(II) complexes with promising anticancer activity |
