# Supplementary material for: Metal based donepezil analogues designed to inhibit human acetylcholinesterase for Alzheimer’s disease
Source: PLoS One. 2019 Feb 20;14(2):e0211935. doi: 10.1371/journal.pone.0211935 (PMC6382135; doi:10.1371/journal.pone.0211935)
Supplement: S4 Table — (DOCX) [file pone.0211935.s004.docx]

**S4 Table**. Selected pharmacokinetic parameter of some Metal Based FDA Approved, Clinical Trials and Promising Drugs

| **Parameters** | **Blood**  **-Brain**  **Barrier** | **Human**  **Intestinal**  **Absorption** | **P-glycoprotein**  **Inhibitor** | **CYP450 2C9**  **Inhibitor** | **Human**  **Ether-a-**  **go-go-**  **Related**  **Gene** | **Acute Oral**  **Toxicity** | **Rat**  **Acute**  **Toxicity**  **(LD50,mol/k**  **g)** |
| --- | --- | --- | --- | --- | --- | --- | --- |
| **Metal Based Drugs** |  |  |  |  |  |  |  |
| **Cisplatin** | +  (0.94) | +  (0.82) | NI  (0.97) | NI  (0.79) | NI  (0.95) | iii  (0.48) | 2.22 |
| **Carboplatin** | +  (0.86) | -  (0.96)) | NI  (0.98) | NI  (0.86) | NI  (0.97) | iii  (0.60) | 2.11 |
| **Oxaliplatin** | +  (0.50) | -  (0.88) | NI  (0.99) | NI  (0.91) | NI  (0.96) | iii  (0.97) | 1.91 |
| **Porfimer Sodium** | -  (0.64) | -  (0.50) | I  (0.59) | I  (0.62) | NI  (0.84) | iii  (0.59) | 2.52 |
| **Melarsoprol** | +  (0.92) | +  (0.99) | NI  (0.90) | NI  (0.69) | NI  (0.71) | iii  (0.64) | 2.43 |
| **Meglumine antimoniate** | -  (0.67) | -  (0.54) | NI  (0.90) | NI  (0.86) | SI  (0.56) | iii  (0.60) | 2.12 |
| **Sodium stibugluconate** | +  (0.77) | -  (0.96) | NI  (0.94) | NI  (0.88) | NI  (0.79) | iii  (0.53) | 2.44 |
| **Sodium aurothiomalate** | +  (0.96) | +  (0.60) | NI  (0.99) | NI  (0.76) | NI  (0.98) | iii  (0.67) | 2.22 |
| **Sodium aurothiosulfate** | +  (0.92) | -  (0.85) | NI  (0.99) | NI  (0.72) | NI  (0.90) | iii  (0.65) | 2.21 |
| **Auranofin** | +  (0.84) | -  (0.71) | NI  (0.79) | NI  (0.80) | NI  (0.93) | iii  (0.60) | 2.71 |
| **Darinaparsin** | +  (0.54) | +  (0.60) | NI  (0.86) | NI  (0.84) | NI  (0.93) | iii  (0.65) | 2.29 |
| **Padeliporfin** | -  (0.65) | -  (0.87) | NI  (0.84) | NI  (0.66) | NI  (0.66) | iii  (0.55) | 2.61 |
| **Ferroquine** | +  (0.88) | +  (0.92) | I  (0.89) | NI  (0.69) | I  (0.74) | iii  (0.53) | 2.72 |
| **BMOV** | +  (0.62) | -  (0.71) | NI  (0.97) | NI  (0.74) | NI  (0.88) | iii  (0.67) | 2.46 |
| **CTC-96** | +  (0.90) | +  (0.94) | I  (0.57) | NI  (0.80) | NI  (0.84) | iii  (0.59) | 2.66 |
| **RAPTA-C** | +  (0.80) | +  (0.82) | NI  (0.52) | NI  (0.68) | NI  (0.63) | iii  (0.50) | 2.80 |

+ =Positive, - = Negative I= Inhibitor, NI =Non-Inhibitor, III = Category III includes compounds with LD50 values greater than 500mg/kg but less than 5000mg/kg.
