# Supplementary figures and images for: Metal based donepezil analogues designed to inhibit human acetylcholinesterase for Alzheimer’s disease
Source: PLoS One. 2019 Feb 20;14(2):e0211935. doi: 10.1371/journal.pone.0211935 (PMC6382135; doi:10.1371/journal.pone.0211935)

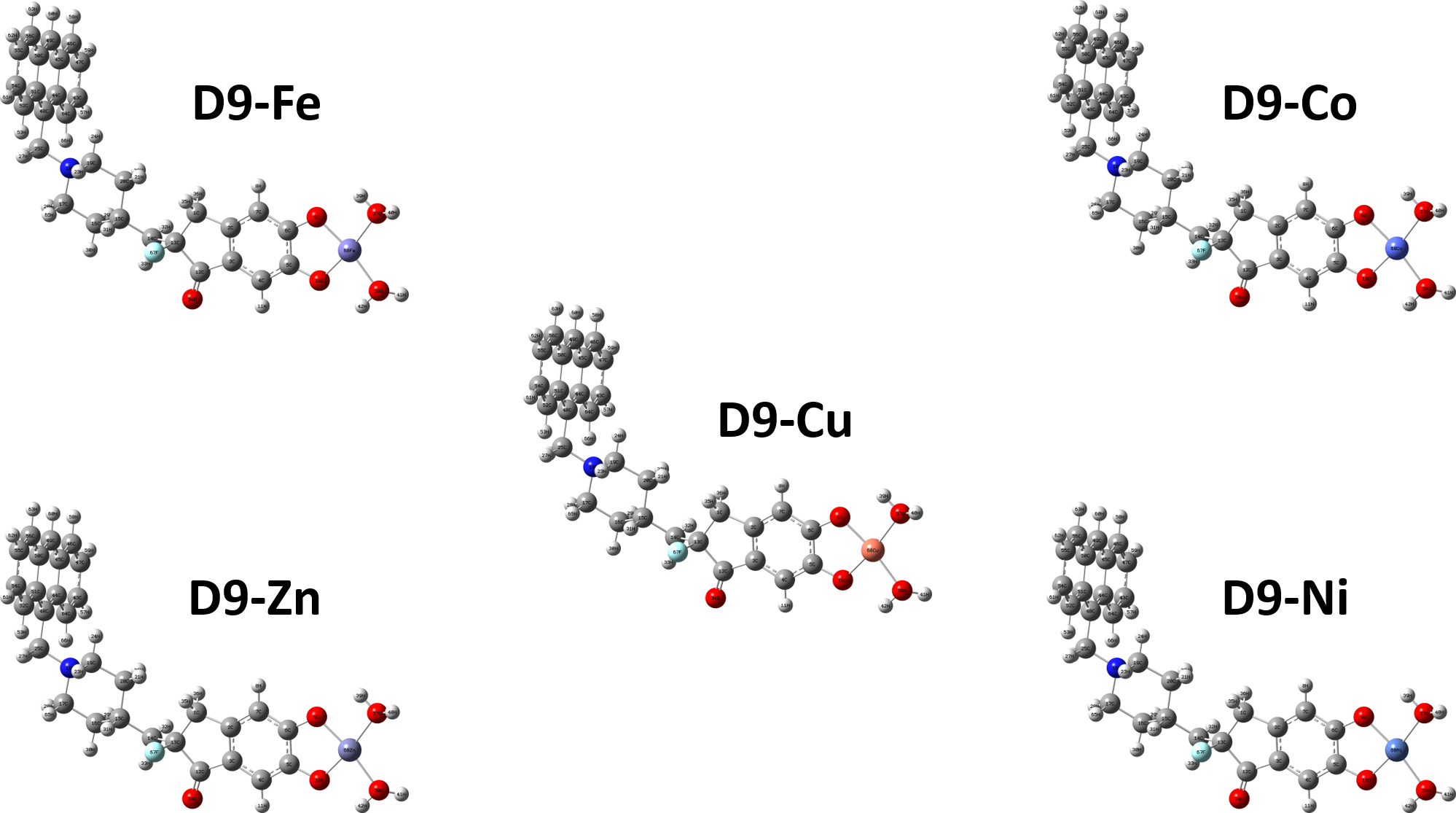

Supplement: S1 Fig — (TIF) [file pone.0211935.s005.tif]

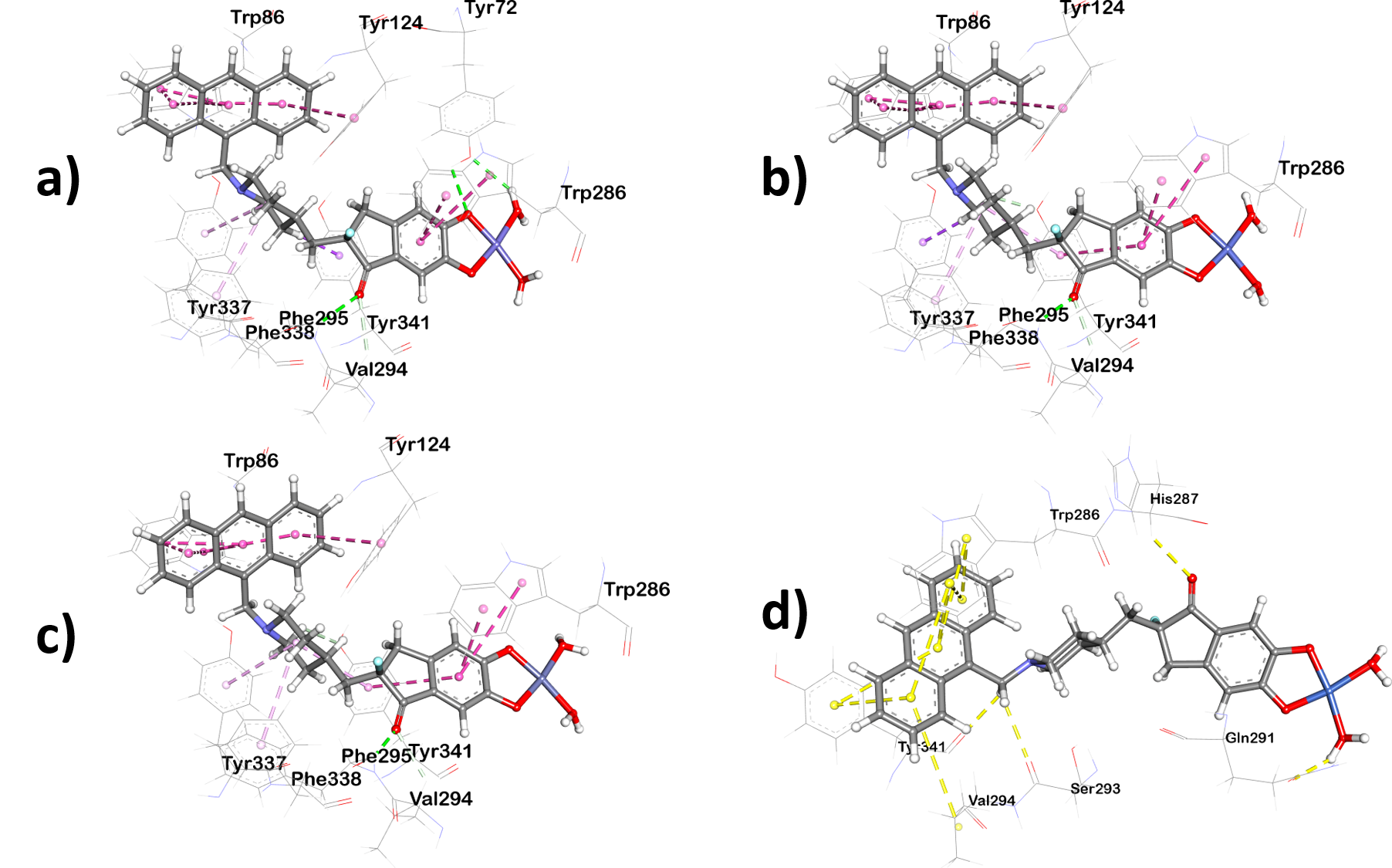

Supplement: S2 Fig — Predicted pose from docking analysis showed the binding orientation map of important amino acids for a) D9-Fe, b) D9-Co, c) D9-Zn and d) D9-Ni, showing hydrogen bond interaction (green color), including π–π stacking (pink color). (TIF) [file pone.0211935.s006.tif]
